# Positive valence music restores executive control over sustained attention

**DOI:** 10.1371/journal.pone.0186231

**Published:** 2017-11-16

**Authors:** Carryl L. Baldwin, Bridget A. Lewis

**Affiliations:** Department of Psychology, George Mason University, Fairfax, VA, United States of America; University of Zurich, SWITZERLAND

## Abstract

Music sometimes improves performance in sustained attention tasks. But the type of music employed in previous investigations has varied considerably, which can account for equivocal results. Progress has been hampered by lack of a systematic database of music varying in key characteristics like tempo and valence. The aims of this study were to establish a database of popular music varying along the dimensions of tempo and valence and to examine the impact of music varying along these dimensions on restoring attentional resources following performance of a sustained attention to response task (SART) vigil. Sixty-nine participants rated popular musical selections that varied in valence and tempo to establish a database of four musical types: fast tempo positive valence, fast tempo negative valence, slow tempo positive valence, and slow tempo negative valence. A second group of 89 participants performed two blocks of the SART task interspersed with either no break or a rest break consisting of 1 of the 4 types of music or silence. Presenting positive valence music (particularly of slow tempo) during an intermission between two successive blocks of the SART significantly decreased miss rates relative to negative valence music or silence. Results support an attentional restoration theory of the impact of music on sustained attention, rather than arousal theory and demonstrate a means of restoring sustained attention. Further, the results establish the validity of a music database that will facilitate further investigations of the impact of music on performance.

## 1 Introduction

Music impacts many aspects of everyday life including our ability to maintain executive control of attention, particularly during sustained attention tasks. Music has sometimes been found to improve performance during difficult vigilance tasks [1,2]; but, in other instances to degrade performance [3]. More recently, Kuhbandner and Zehetleitner [4] found that tempo and valence impacted different aspects of executive attentional control. Jiang, Scolaro, Bailey, and Chen [5] observed that negative valence music enhanced processing of attentional cues while positive valence music had no effect. And, Pereira et al. [6] found differential activation of specific brain regions as a function of music that is familiar versus unfamiliar and liked versus disliked.

A major difficulty with comparing results across these studies is that a wide variety of different types of music have been used and this music has varied in valence, tempo, familiarity, and personal preference. For example, Davies et al. used instrumental music that people reported generally finding stimulating, but not necessarily liking and they were unlikely to be familiar with it. Pereira et al. used pop/rock music that people were likely to be familiar with. In order to progress in our understanding of how music impacts executive control of attention, normed databases will need to be established that allow the separation of key characteristics, such as valence, tempo, familiarity, and preference. Understanding methods of facilitating executive control during vigilance tasks is important due to their sheer prevalence in today’s society.

Many industrial and military tasks require people to pay vigilant attention for sustained periods of times (e.g., radiologists, operators of unmanned aerial vehicles, nuclear power plant engineers, baggage screeners, etc.). As over 50 years or vigilance research has shown, maintaining vigilant attention is extremely difficult and stressful [7]. Typically, after some period of time that ranges from 5 to 15 minutes [7] performance decrements occur in the form of either increased response time, decreased accuracy, decreased perceptual sensitivity or some combination of these metrics [8–13]. These performance declines are referred to as the vigilance decrement.

A leading alternate theory of the mechanism underlying the vigilance decrement implies cognitive underload or mindlessness [14,15] as opposed to the “resource theory” explanation [10,11,16–18] which theorizes that the vigilance decrement results from depletion of cognitive resources. According to the mindlessness theory, with increased time on task, people lose their ability focus on the task due to failure or weakening of the supervisory attention system [14,19]. In support of this theory, Manly et al. found that scores on the Cognitive Failures Questionnaire, a questionnaire designed to assess absent-mindedness, was correlated with errors in the Sustained Attention to Response Task (SART) developed by Robertson and colleagues [15]. In this view, attentional resources are withdrawn either purposefully, such as in the case of Motivational Control Theory [20]or absentmindedly due the routinized nature of the task [14,15].

However, scant attention has been placed on potential methods of reducing performance decrements using SART paradigms. This was one aim of the present investigation. Assuming that SART tasks are resource depleting and stressful, we asked what type of break might best restore attentional resources. In other words, if someone has to perform an unpleasant task requiring frequent responses of relatively low demand for longer than they would care to, is there some type of break that might help them restore their executive control? What type of break would be most likely to restore attentional reserve in order to allow them to continue performing the task again without much performance decrement? Music is frequently used as means of promoting relaxation. Could the everyday task of listening to popular music facilitate the restoration of attentional resources, and if so, what kind?

Of the models used to explain the high levels of reported workload found in traditional vigilance paradgims, two of the more frequently studied, competing models are the direct-cost model which posits that workload is increased due to the demanding nature of the task [21,22] and the indirect cost model which posits that workload is increased due to boredom inherent in traditional vigilance paradigms [23]. A study by Hitchock et al. [21] tested one aspect of the model: whether reducing the difficulty of the task would reduce perceived workload, by adding reliable cues prior to targets and found that the decrease in the difficulty of the task did decrease workload ratings but did not decrease overall boredom. In order to further strengthen the direct-cost model, Alikonis et al. [3] used music to stimulate observers during a vigilance task. Alikonis et al. found that the addition of music during the task did not lower perceptions of workload, but did lower boredom. However, they found that the addition of music during the task also decreased observer sensitivity scores, pointing out that music may have acted as a distractor during their study.

Listening to comforting music has been shown to increase engagement of the default mode network [24]. Though the specific role of the default mode network is still a matter of debate, it is suggested that it may provide a means of resting or relaxing the executive control system. It follows then that some types of music, in particular relaxing music (positive valence slow tempo music) may promote engagement of the default mode network and thereby provide a restorative environment following the resource depletion associated with a sustained attention task.

### 1.1 Attentional restoration theory

Kaplan [25] developed the Attention Restoration Theory. According to this theory, recovery from the fatigue induced by directed attention requires spending time in environments that are different from those inducing the fatigue. In particular, the environment should be distinct or physically removed from the fatiguing task environment. The restorative environment should promote one’s ability to hold attention effortlessly and have a scope and coherence to allow engagement that is compatible with one’s inclinations. These environments are considered restorative in that they allow fatigued persons to rest internal mechanisms required to maintain directed attention giving time to restore directed attentional capacity.

Restorative environments may facilitate engagement of the default mode network. According to a leading theory of the role of attention and default mode network, referred to as the decoupling hypothesis,[26] engagement of the default mode network is associated with reduced processing of sensory information in the external environment. Attentional restoration theory may complement the hypothesis to the extent that restorative environments engage the default mode network. Engagement of the default mode network might allow the restoration of cognitive resources depleted during focused task performance.

Berto [27] utilized the SART to elicit a vigilance decrement in a very short (less than 10 minutes) amount of time to show the effect of restorative environments on directed attention. In Berto’s study, participants completed the SART, followed by an intervention in which subjects were shown pictures of either built-up (urban) environments or natural environments followed by a second SART block. Berto found that participants who were shown natural environments performed significantly better than control participants on the second block of the SART.

In the present investigation, we reasoned that providing a break consisting of popular music of positive affect would provide a restorative environment capable of replenishing attentional reserve in between SART vigils. Listening to music is discreetly different from performing a SART and positive affect music can be reasoned to allow effortless non-directed attention. We hypothesized that positive affect music of a slow tempo would be particularly effective in restoring resources since it would facilitate decoupling or resting of the mechanisms required by focused sustained attention. We examine this hypothesis in Experiment 2. However, in the first study we first sought to establish norms for positive and negative valence music of slow and fast tempo. Numerous previous studies have attempted to examine music of these types but normed databanks of music are sparse. Even fewer (if any) studies have established music repositories of these categories using popular music. Therefore, in Experiment 1 we sought to establish repositories of popular music in each category that could be used in further research.

## 2 Experiment 1

The goal of this study to create and validate a database of currently popular songs categorized based on both their valence and their speed. Researchers chose popular songs that were highly familiar and varied in both valence and tempo. The set of songs was then presented to an independent sample of listeners who were asked to rate each song using 5-point likert scales for the dimensions valence and speed and to complete a shortened Geneva Emotional Music Scale (GEMS-9: 27) for each song.

### 2.1 Methods

#### 2.1.1 Pre-testing

Prior to testing, the researchers identified 80 popular songs that varied on both valence (songs considered by the researchers to be positive or negative) and speed (songs considered fast or slow). Note that several previous studies have found a strong association between major and minor modes and positive and negative perceived affect, respectively [28,29,20]. Here we use the term valence as perceived emotion, and not musical mode per se. These songs were then sorted by the researchers into 4 groups of 20 songs each: positive fast songs, positive slow songs, negative fast songs and negative slow songs.

#### 2.1.2 Participants

Participants were 69 (21 male, average age 21.59 years) undergraduate and graduate students recruited through the University’s psychological research participation system. Participants were compensated with a small amount of research credit for their participation in the study.

#### 2.1.3 Stimuli and apparatus

Songs were chosen based on their appearance on social media and in current “top hits” lists. Clips were created using Adobe Audition CS6, from the main “hook”, or refrain, of the song, lasting from 12 s to 49 s, based on the length of the main hook line. All song clips included a short fade in and out to eliminate clipped beginnings or endings. Songs were equated for perceived loudness in Adobe Audition CS6, however, participants were allowed to listen to songs at whatever volume they preferred. Participants completed the study on their personal computers, remotely.

#### 2.1.4 Procedure

Participants signed up for the study via the online research participation system, whereby they were then given access to an online survey through Qualtrics survey software. Participants gave consent and then completed a test of their volume, during which they were asked to adjust their system volume such that they could hear a sound clip. Participants were asked to verify the word played in the sound clip before continuing to ensure their system was at an adequate volume. Participants then completed a block of demographic and music experience-related questions. After completion of the demographic block, participants were presented four randomized blocks of 20 song clips (also randomized) and asked to listen to the full song, and then to rate the song using a modified version of the GEMS-9 (Table 1).

**Table 1:** GEMS-9 ratings scale (29).

Feelings	Descriptions
Wonder	Filled with wonder, Dazzled, Moved
Transcendence	Fascinated, Overwhelmed, Feelings of Transcendence and spirituality
Power	Strong, Triumphant, Energetic
Tenderness	Tender, Affectionate, In love
Nostalgia	Nostalgic, Dreamy, Melancholic
Peacefulness	Serene, Calm, Soothed
Joyful Activation	Joyful, Amused, Bouncy
Sadness	Sad, Sorrowful
Tension	Tense, Agitated, Nervous

The GEMS-9 uses a 5-point likert scale (“Not at all”, “Somewhat”, “Moderately”, “Quite a lot” and “Very Much”) to evaluate the emotions inspired by music. Each song was also rated on a 5-point scale on liking, speed, (from “Very Slow” to “Very Fast”), valence (from “Very Negative” to “Very Positive”) and familiarity. Participants were given as much time as they wanted to complete the survey.

### 2.2 Results

**Fig 1:**
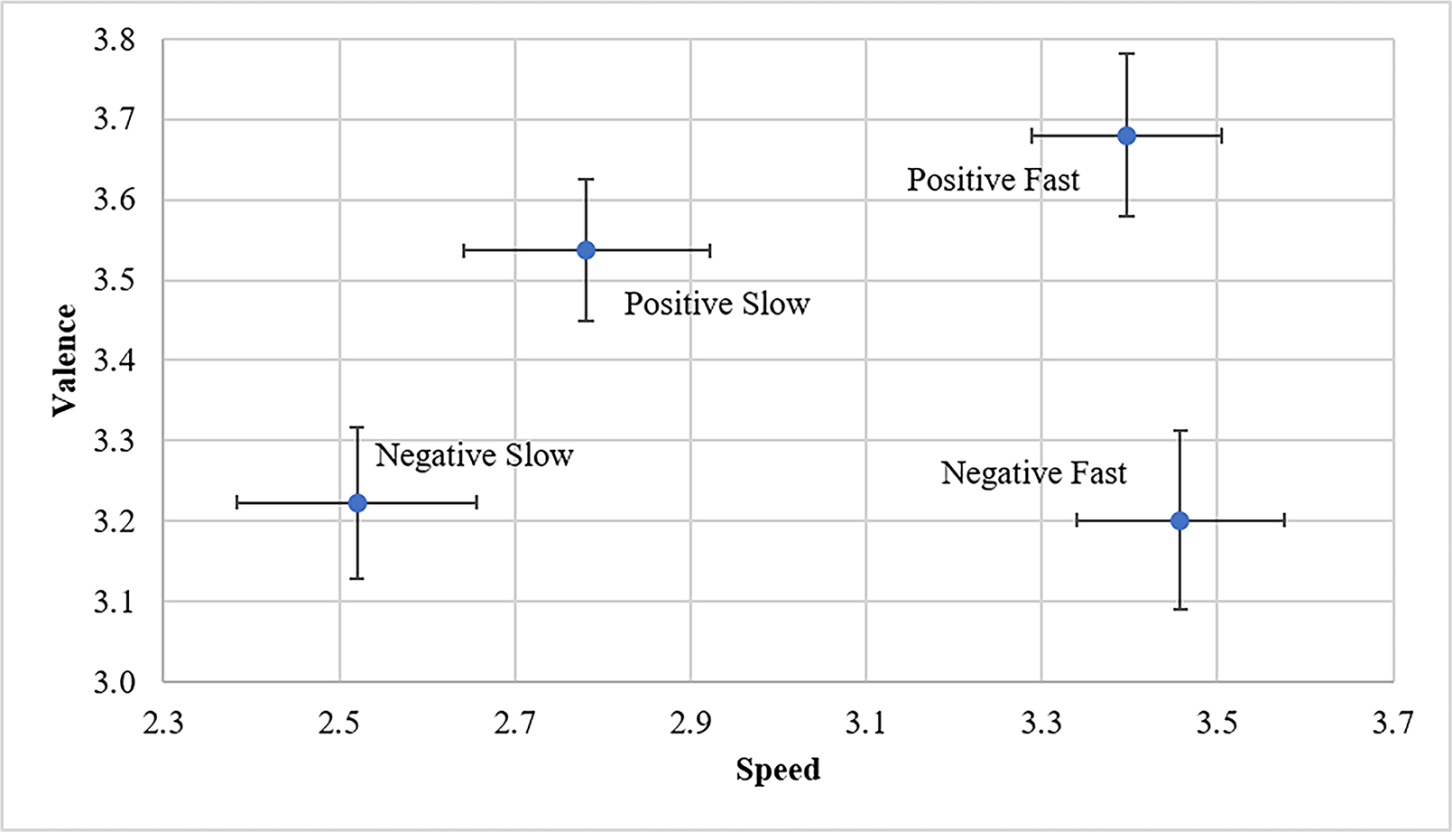
Average valence and speed ratings by group. Error bars indicate standard error.

A two (valence: positive and negative) by two (speed: fast and slow) analysis of variance (ANOVA) for the dependent variable speed rating revealed significant main effect, F(1,76) = 37.76, p < .001. Specifically, the fast song groups were rated significantly faster than slow song groups. (See Fig 1). Post hoc analysis indicate that there were no significant valence differences between positive fast and positive slow song groups. Though analyses did indicate that the positive slow songlist (M = 3.54, SD = 0.39) was not significantly more positive than either negative group (negative fast: M = 3.20, SD = 0.50 and negative slow: M = 3.22, SD = 0.42) despite being rated somewhat more positively. There was not a significant interaction between valence and speed.

A two (valence: positive and negative) by two (speed: fast and slow) ANOVA for the dependent variable valence rating revealed a significant main effect, F(1,76) = 15.98, p < .001, such that positive song groups were rated significantly more positively than negative song groups (Fig 1). Post hoc analysis indicate that there were no significant speed differences between negative fast and negative slow groups, nor were there significant speed differences between negative fast and positive fast groups nor negative slow and positive slow. There was not a significant interaction between valence and speed.

**Fig 2:**
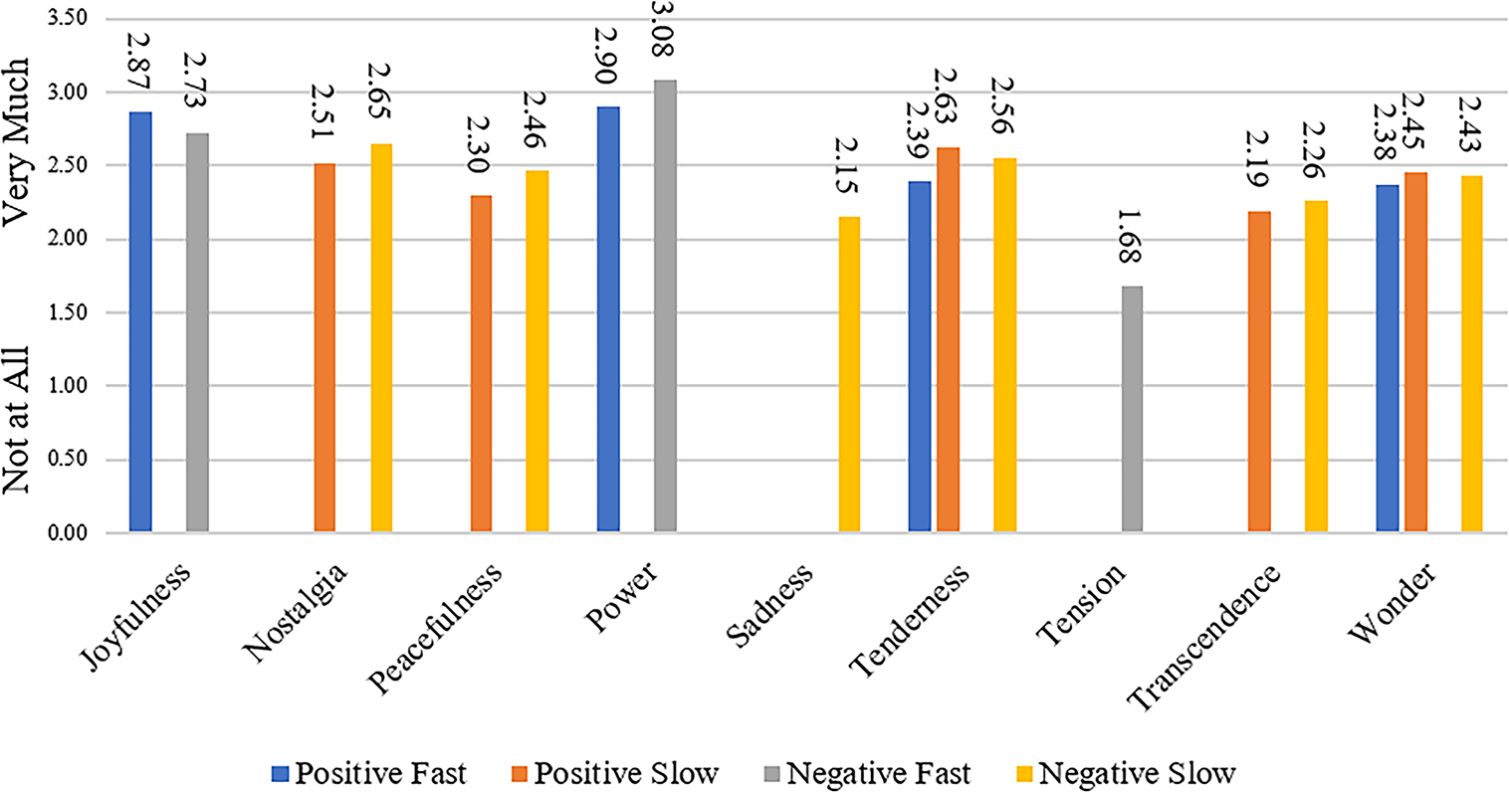
Emotionality ratings by group. Bars represent the highest value for each emotion by group, where bars present are generally higher than those not shown.

Analysis of GEMS data indicated significant effects of all emotions by group, such that songs in the positive fast category were rated as generally more joyful than those in other groups. Songs in the positive slow category showed higher levels of nostalgia and tenderness. Songs in the negative fast category showed higher levels of power and tension and songs in the negative slow category showed higher levels of nostalgia, peacefulness, sadness and tenderness (Fig 2). There were no significant differences in liking or familiarity by group.

### 2.3 Discussion of experiment 1

The goal of Study 1 was to create and validate a set of currently popular music that varied systematically on perceptions of both valence and speed. Results indicate that positive slow and positive fast songs were rated as significantly more positive than were negative slow and negative fast songs, and that positive fast and negative fast songs were rated as significantly faster than were positive slow and negative slow songs. Positive fast songs were rated as the most joyful songs, positive slow songs were described very similarly to negative slow songs, though negative slow songs were the only group rated more highly on sadness. Negative fast songs were the only songs with higher tension ratings.

These results indicate that it is possible to create lists of popular songs that are rated significantly differently based on both valence and speed. These repositories of music were established to be suitable for examining the second major aim of this investigation, namely can music of positive valence restore focused attention resources?

## 3 Experiment 2

In order to validate the results of Experiment 1, a second study was designed. The goal of Experiment 2 was to assess the effect of music presentation in mitigating fatigue in a sustained attention task. It was hypothesized that music would be better than a break alone but that positive valence music, in particular, would facilitate positive mood, which would subsequently be associated with greater stress reduction and more attentional restoration.

### 3.1 Methods

#### 3.1.1 Participants

Participants were 89 (24 male, average age 20.73 years) graduate and undergraduate students recruited through the University’s psychological research participation system. Participants were compensated with a small amount of research credit for their participation in the study.

#### 3.1.2 Stimuli and apparatus

Experiment 2 was run in a sound attenuated lab room on a desktop computer. The experimental task was presented in SuperLab 4.5. The experimental task presented was a Go-NoGo task referred to as the Sustained Attention to Response Task (SART: see 14,27). This version of the task involves the rapid presentation of black numbers on a white background. Numbers are presented for 250 ms with a 750 ms response window. Participants can respond at any time beginning when the number is presented until the response window ends and the next number is presented. Numbers presented were 0–9. Participants were required to respond with a mouse click to every number except for the number 3 (a NoGo trial). Each number was presented 42 times for a total of 420 trials in each 7-minute block.

This task varies from the traditional vigilance paradigm in response encoding. We chose to adhere to the codes used by Dillard et al. [16]. Specifically, as shown in Table 2, non-responses to targets (the number 3) were coded as hits, whereas responses to targets were coded as misses. Responses to non-targets (all other numbers) were coded as correct rejections and non-responses to non-targets were coded as false alarms.

**Table 2:** Response coding.

	Target	Non-target
Response	Miss	Correct Rejection
Non-response	Hit	False Alarm

Music included for intervention groups consisted of 15 songs from each previously evaluated group such that each intervention playlist lasted approximately 7 minutes.

#### 3.1.3 Procedure

All procedures were approved by George Mason University’s Institutional Review Board. Participants first gave written informed consent for the protocol and then were given instructions for the SART task. After receiving instructions, participants completed a short (less than 2 minutes) practice SART task. Once participants indicated they were comfortable with the task they completed the experimental session. The experimental session consisted of a 7 minute SART block, followed by an intervention (varying based on group), followed by a second 7 minute SART block. Groups included four music interventions (consisting of about 7 minutes of positive fast, positive slow, negative fast and negative slow music clips), and two control groups: one group received no music, but did have a 7-minute break in which they were asked to relax and sit in silence, and one group who received no break and immediately began the second SART block. After completing the experimental session participants completed a short demographic survey, during which they were asked questions about their musical experience and history and, for those in a musical condition, were asked to re-listen to their playlist and rate the overall playlist using the same modified GEMS scale as in Experiment 1.

### 3.2 Results

Results indicate no significant effects of age or gender nor significant differences in gender or age by condition. Variables of interest included delta miss rate calculated as the percentage of misses (or times that a participant clicked on a 3) in block 1 subtracted from the percentage of misses in block 2, where negative numbers would indicate a decrease in misses after the intervention and a positive number would indicate an increase in misses. This variable is directly related to d-prime where a decrease in delta miss rate is related to an increase in d-prime or the sensitivity of the participant in detecting the target.

**Fig 3:**
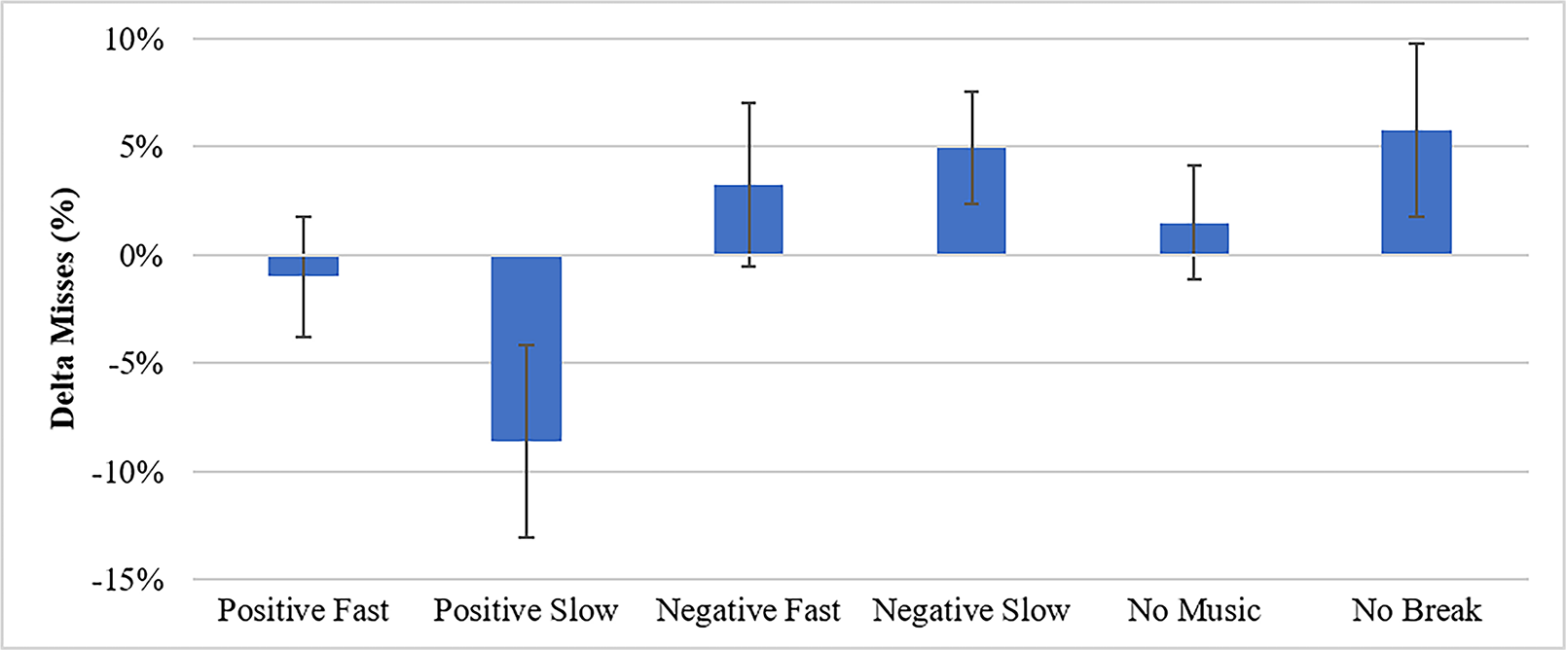
Delta miss percentages by condition.

Results indicate a significant effect of condition on delta miss rate, F(5,83) = 2.43, p = .042, where subjects in the positive conditions and particularly the positive slow condition showed significant reductions in misses after the intervention but subjects in negative conditions or no break conditions showed significant increases in misses after the intervention (Fig 3 and Table 3). There was no significant difference in delta correct rejection rate indicating that the change in delta miss rate was not due simply to a change in response bias (or participants responding to more stimuli in general).

**Table 3:** Delta miss percentages as a function of condition.

Condition	Mean	Std. Deviation
Positive Fast	-1%	11%
Positive Slow	-9%	17%
Negative Fast	3%	15%
Negative Slow	5%	10%
No Music	2%	10%
No Break	6%	14%

**Fig 4:**
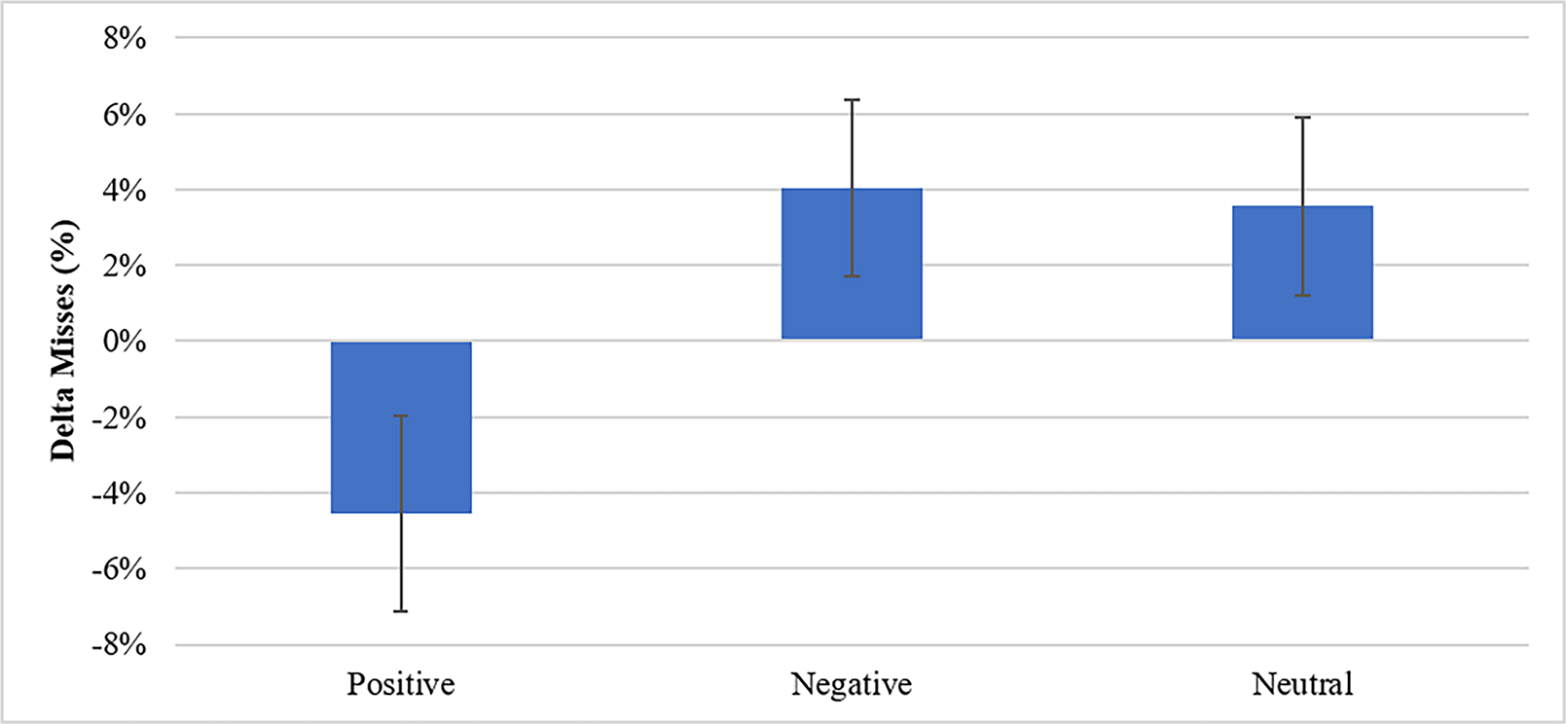
Delta miss percentages by intervention valence.

This effect is more pronounced when discussed in terms of the effect of overall valence, F(2,86) = 4.21, p = .018, where participants who received an intervention consisting of a playlist with a positive valence saw no decrement after the intervention but actually saw an overall decrease in misses relative to no intervention or an intervention with songs having a negative valence (Fig 4). There was no significant difference in delta correct rejection rate.

**Fig 5:**
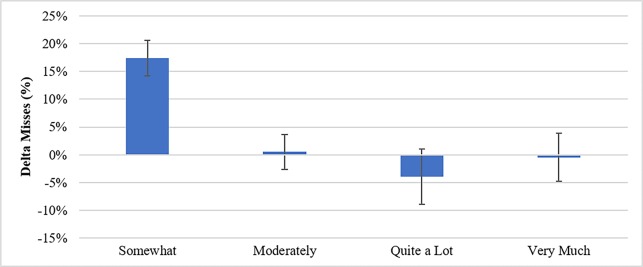
Delta miss percentages by ratings of liking for musical interventions.

Furthermore, when song preference, or “liking” is taken into account, participants who did not like their intervention playlist had significantly more misses than those who at least liked their songs moderately or more (where possible “liking” included “Not at all”, “Somewhat”, “Moderately”, “Quite a lot” and “Very Much”), F (3,38) = 3.24, p = .03 (Fig 5).

Results indicate that, where participants generally liked their intervention playlist, variation in delta miss rate was more consistent with overall valence effects, but for participants who did not generally like their intervention playlist, valence did not make a difference: all participants showed increased delta miss rate.

#### 3.2.1 Predictive power assessment

In addition to analysis of general rates of accuracy, positive and negative predictive powers were assessed as in Dillard et al. [16]. Positive predictive power (PPP) is defined as the proportion of responses to the target (hits) are correct where negative predictive power (NPP) is defined as the proportions of responses indicating the absence of a target (correct rejections) are correct [16]. The equations used in the calculation of PPP and NPP are presented below.

$$PPP=\frac{Hits}{\left(Hits+False\;Alarms\right)}$$

$$NPP=\frac{Correct\;Rejections}{\left(Correct\;Rejections+Misses\right)}$$

Neither PPP nor NPP by condition were found to be significant, however, while non-significant (F(2,86) = 2.37, p = .1) trends towards increased PPP for participants receiving positive valence music (as measured by delta PPP or PPP for block 1 subtracted from PPP for block 2) were observed.

### 3.3 Discussion of experiment 2

The goal of Experiment 2 was to assess the effect of varying music types on fatigue mitigation in a sustained attention task. It was found that only positive music mitigated misses, where negative music actually tended to increase misses above the no music condition, though not above the no break condition. Additionally, and importantly, music preference had a differential effect on misses where those who liked the music less (“Somewhat” being below the midline of the preference likert scale) tended to have increased misses no matter which group they were in, while those who at least moderately like the music showed trends more similar to the general effects on misses based on their group.

## 4 General discussion

Tasks that become routine (such as a daily commute, or baggage screening) may require executive control over attentional resources in order to prevent distractions from external or internal sources (e.g., texting or mind wandering, respectively). According to the Motivational Control Theory [30], seeking alternative forms of engagement may even serve some evolutionary purpose. Actively trying to maintain sustained attention is hard work [10]. For routine, automatized tasks it may actually be to our benefit to let our minds drift to some more interesting topic in order to stay alert, if not engaged and focused on the primary task at hand. This issue is currently under active debate and we make no pretense of resolving that debate here. However, the fact that music of positive valence, particularly of slow tempo, restored vigilant attention more than no break or arousing music of fast tempo suggests that attentional resources need to be replenished. If the SART simply became routine, then any type of break should improve performance. Results of the current investigation do not support this theory. Additionally, music that was preferred by participants had positive effects on performance while music that was disliked had significantly more negative effects on performance, regardless of the type of music. This finding may be important in explaining other findings where music may have shown negative effects or distraction to participants.

Further, the present investigation established normed repositories of music in four distinct categories varying in affect and tempo. Specifically, popular music pieces of fast and slow tempo with positive valance and fast and slow tempo with negative valence were established. These normed repositories will be made available upon request. They are free and open to researchers for all types of noncommercial use.

In sum, music influences both cognition and performance and as illustrated in the current investigation, music of specific types can be used to restore attentional reserve.

## Supporting information

S1 File (SAV)
